# *In vivo* Whole-Cell Recordings Combined with Electron Microscopy Reveal Unexpected Morphological and Physiological Properties in the Lateral Nucleus of the Trapezoid Body in the Auditory Brainstem

**DOI:** 10.3389/fncir.2016.00069

**Published:** 2016-08-24

**Authors:** Tom P. Franken, Philip H. Smith, Philip X. Joris

**Affiliations:** ^1^Laboratory of Auditory Neurophysiology, Department of Neurosciences, Katholieke Universiteit LeuvenLeuven, Belgium; ^2^Department of Neuroscience, University of Wisconsin School of Medicine and Public HealthMadison, WI, USA

**Keywords:** Mongolian gerbil, LNTB, patch clamp, phase-locking, ITD, LSO, mLNTB, pvLNTB

## Abstract

The lateral nucleus of the trapezoid body (LNTB) is a prominent nucleus in the superior olivary complex in mammals including humans. Its physiology *in vivo* is poorly understood due to a paucity of recordings. It is thought to provide a glycinergic projection to the medial superior olive (MSO) with an important role in binaural processing and sound localization. We combined *in vivo* patch clamp recordings with labeling of individual neurons in the Mongolian gerbil. Labeling of the recorded neurons allowed us to relate physiological properties to anatomy at the light and electron microscopic level. We identified a population of quite dorsally located neurons with surprisingly large dendritic trees on which most of the synaptic input impinges. In most neurons, one or more of these dendrites run through and are then medial to the MSO. These neurons were often binaural and could even show sensitivity to interaural time differences (ITDs) of stimulus fine structure or envelope. Moreover, a subpopulation showed enhanced phase-locking to tones delivered in the tuning curve tail. We propose that these neurons constitute the gerbil main LNTB (mLNTB). In contrast, a smaller sample of neurons was identified that was located more ventrally and that we designate to be in posteroventral LNTB (pvLNTB). These cells receive large somatic excitatory terminals from globular bushy cells. We also identified previously undescribed synaptic inputs from the lateral superior olive. pvLNTB neurons are usually monaural, display a primary-like-with-notch response to ipsilateral short tones at CF and can phase-lock to low frequency tones. We conclude that mLNTB contains a population of neurons with extended dendritic trees where most of the synaptic input is found, that can show enhanced phase-locking and sensitivity to ITD. pvLNTB cells, presumed to provide glycinergic input to the MSO, get large somatic globular bushy synaptic inputs and are typically monaural with short tone responses similar to their primary input from the cochlear nucleus.

## Introduction

The SOC is a collection of brainstem nuclei concerned primarily with auditory processing. Two principal nuclei, the medial and lateral superior olive (MSO and LSO), are crucial components of sound localization circuitry. They are surrounded by a series of nuclei designated periolivary (Warr, 1966; Guinan et al., 1972b; Tsuchitani, 1977; Spirou and Berrebi, 1996; Kulesza, 2007, 2008). The periolivary area immediately ventral to LSO and lateral to MSO has been defined as the LNTB and is found in all mammals studied including human (Kulesza, 2008). Little is known about the LNTB, in terms of morphology, connectivity, and physiology. MSO neurons are sensitive to time differences in the arrival of sound at the two ears (ITDs) by a process akin to coincidence detection on inputs from each side (Yin and Chan, 1990; Franken et al., 2015). The MSO not only receives excitatory but also inhibitory inputs relayed from each ear (Cant and Hyson, 1992; Grothe and Sanes, 1993; Smith, 1995), and the LNTB is thought to be the source of ipsilateral inhibition (Cant and Hyson, 1992; Kuwabara and Zook, 1992; Roberts et al., 2014). The function of inhibition on MSO cells has been the subject of intense debate (Brand et al., 2002; Joris and Yin, 2007; Pecka et al., 2008; Roberts et al., 2013; Myoga et al., 2014; Franken et al., 2015).

Extracellular recordings from the LNTB area in the cat suggested that most cells are monaural, responding only to sounds presented to the ipsilateral ear (Guinan et al., 1972a,b; Tsuchitani, 1977). Short tone responses of units recorded in this area generated primary-like, primary-like-with-notch, chopper, onset or phase-locked PSTHs. Anatomically the cat LNTB has been divided into three sub-regions based on light and electron microscopic evaluation of cytological characteristics, neurochemistry and patterns of innervation (Spirou and Berrebi, 1996, 1997; Spirou et al., 1998). These regions are designated the main (mLNTB), posteroventral (pvLNTB) and hilar (hLNTB) subdivisions. Cells in pvLNTB are glycinergic, resemble bushy and MNTB cells in terms of their cell body morphology, and receive large excitatory terminals on their cell bodies presumably from GBCs. Cells in mLNTB and hLNTB show a variety of cell body morphologies, typically do not heavily label with glycine antibodies and do not receive large somatic terminal inputs. Besides the MSO, the cochlear nucleus and in some cases also the inferior colliculus (IC) have been described as projection targets of the LNTB (Adams, 1983; Spangler et al., 1987).

Despite the recent interest in the potential role of the LNTB in low-frequency sound-localization circuitry, there is no experimental evidence regarding the synaptic or spike responses of any labeled LNTB cell to auditory stimuli in any species. Furthermore, it is unclear how the parcellation of LNTB neurons described in the cat relates to physiology or projection patterns, and whether a similar subdivision exists in other species such as gerbils, which have often been used in recent studies of the SOC (Pecka et al., 2008; van der Heijden et al., 2013; Roberts et al., 2014; Franken et al., 2015). Therefore it is important to determine (1) whether different LNTB subdivisions can be anatomically defined in the gerbil; (2) whether these have different physiological properties, and (3) whether the sound-driven responses of LNTB cells show phase-locking, as required for their proposed role in the generation of ITD-sensitivity in MSO (Myoga et al., 2014), although recent evidence suggests that glycinergic inhibition does not contribute to best ITD in the MSO (Franken et al., 2015).

To this end we have recorded intracellular responses of cells in the gerbil LNTB to auditory stimuli, labeled the recorded cells with biocytin, and evaluated the location and anatomical properties of these cells at the light and electron microscopic level.

## Materials and Methods

### Animals and Preparation

All procedures were approved by the KU Leuven Ethics Committee for Animal Experiments. We used adult and juvenile (P29–P31) Mongolian gerbils of either sex. The animals were brought under general anesthesia by injecting a mixture of ketamine (80–120 mg/kg) and xylazine (8–10 mg/kg) in NaCl 0.9% i.p. Maintenance of anesthesia was ensured by additional doses of ketamine (30–60 mg/kg) and diazepam (0.8–1.5 mg/kg) in aqua i.m., guided by depth of anesthesia judged by the toe pinch reflex. Body temperature was kept at 37°C by a homeothermic blanket (Harvard Apparatus) and a heating lamp positioned above the animal. The ventrolateral brainstem was exposed as described previously (Franken et al., 2015). The pinnae folds around the external acoustical meatus were removed and a transbulla craniotomy was made on the left side of the brainstem. The contralateral bulla was also opened to maintain acoustic symmetry. Before electrode penetration, the meningeal layers were removed.

### Electrophysiology

Patch clamp electrodes were pulled from borosilicate glass capillaries (1B120F-4, World Precision Instruments, Inc., Sarasota, FL, USA) with a horizontal puller (Model P-87, Sutter Instrument Co.). The electrode resistances were 5–8 MΩ when filled with the solution. The internal solution contained 115 mM K gluconate (Sigma), 4.42 mM KCl (Fisher), 10 mM Na_2_ phosphocreatine (Sigma), 10 mM HEPES (Sigma), 0.5 mM EGTA (Sigma), 4 mM Mg-ATP (Sigma), 0.3 mM Na-GTP (Sigma) and 0.1 or 0.2% biocytin (Invitrogen), with pH 7.30 (adjusted with KOH, Sigma) and osmolality 300 mmol/kg (adjusted with sucrose, Sigma) (Roberts et al., 2014). Patch clamp recordings were obtained using the blind *in vivo* method as described before (Margrie et al., 2002; Franken et al., 2015). Membrane potential recordings were obtained in current clamp using a patch clamp amplifier (BVC-700A; Dagan, Minneapolis, MN, USA). The analog signal was low-pass filtered (cut-off frequency 5 kHz), digitized at 50–100 kHz and saved using scripts in MATLAB (The Mathworks) or IgorPro (WaveMetrics). Series resistance was 51.7 ± 10.8 MΩ (mean ± SEM; *N* = 8; excluding one outlier with a series resistance >100 MΩ). Initial resting membrane potential was –54.6 ± 1.95 mV (mean ± SEM; *N* = 10).

### Stimuli

The experiments were performed in a double-walled sound-proof booth (IAC, Niederkrüchten, Germany). TDT System II hardware controlled by MATLAB scripts was used to generate and present sound stimuli. Etymotic speakers attached to hollow ear bars delivered the sound stimuli to the ears. Before each experiment, the stimulus system was acoustically calibrated with a probe microphone (Bruel and Kjaer, Nærum, Denmark). When intracellular access was obtained, frequency-tuning was studied using a threshold-tracking algorithm during monaural or binaural short tone presentation. The triggering was usually set for action potentials but was occasionally set for subthreshold events. We then collected responses to monaural tones varied over a range of frequencies (isolevel datasets; typical settings: 50–309 Hz to 2000–30000 Hz in steps of 0.3 octave or 50 Hz, tone duration 50–250 ms, interstimulus interval 200–300 ms, 60 or 70 dB SPL, 1–20 repetitions). In addition, we presented monaural short tones at CF ipsilaterally and contralaterally over a range of SPLs (isofrequency datasets; typical settings: tone duration 50 or 100 ms, interstimulus interval 150 or 200 ms, sound levels from 10 to 80 or 90 dB in steps of 10 dB, 5–200 repetitions). Sometimes such monaural isofrequency datasets were obtained for other frequencies as well. For some neurons, ITD-sensitivity to fine-structure (the instantaneous pressure fluctuations of the sound waveform) was evaluated using binaural beats (binaural tones with a small frequency difference in each ear so that the interaural phase difference varies continuously (Kuwada et al., 1979); typical parameters: 5000 ms long, interstimulus interval 6000 ms, 1 Hz beat frequency) and ITD-sensitivity to envelope (slower changes in amplitude of the sound waveform) was evaluated using amplitude-modulated tones at CF with a 1 Hz beat between the modulation envelopes at the two ears (Joris and Yin, 1995).

### Analysis

We wrote scripts in MATLAB (The Mathworks) and IgorPro (WaveMetrics) to analyze the data. Membrane potentials were corrected for the junction potential by subtracting 10 mV from the measured potential (Roberts et al., 2014). Steady-state and peak input resistances were derived from voltage responses to hyperpolarizing current steps by calculating, respectively, the median membrane potential during the last 10% of the step and the minimal membrane potential during the step response. Membrane time constants were derived by fitting an exponential function to hyperpolarizing current responses and calculating the average time constant to the two or three smallest responses with a good fit. A regularity analysis was performed on the spike responses to monaural tones at CF, using the method of (Wright et al., 2012). To determine the precision of phase-locking, we used VS (Goldberg and Brown, 1969). Only events occurring during the stimulus were included, but the onset response (first 10 ms) was discarded (Joris et al., 1994a). VS was calculated after pooling action potentials across repetitions for the same stimulus parameters within a given isofrequency or isolevel data set. ITD sensitivity was evaluated by treating all spikes during a binaural beat stimulus (discarding the first beat cycle) as unit vectors with an angle corresponding to the phase when the spike occurred in the beat cycle. Best ITD was defined as the angle of the vectorial sum of these unit vectors (Yin and Chan, 1990). ITD functions were smoothed by convolution with a 5-point Hanning window (MATLAB function *hanning*). Statistically significant phase-locking and ITD-tuning was defined as Rayleigh test α ≤ 0.001 (Ruggero and Rich, 1983; Franken et al., 2015).

### Histology and Electron Microscopy

At the end of experiment, the animal was overdosed with pentobarbital and perfused through the heart with saline followed by PFA 4% in 0.1M PO_4_ buffer or PFA 1%/glutaraldehyde 1% and PFA 2%/glutaraldehyde 1%. Tissue processing methods for light and EM have been described previously (Smith et al., 2005, 2010) and are briefly summarized here. The brain was removed and stored refrigerated in PFA 1%/glutaraldehyde 1% for 24 h or longer. The 70-μm thick sections of the brainstem were then cut with a vibratome and the biocytin tracer visualized using the DAB-nickel/cobalt intensification method (Adams, 1981). Sections were rinsed in phosphate buffer and these free-floating sections were inspected with a light microscope to determine the location of the labeled cell, its axon and dendritic tree.

Some of the sections containing the labeled cell body and relevant portions of its dendritic tree and axon were selected to be processed for EM. Sections not selected for EM were mounted on slides, dehydrated, Nissl-stained with cresyl violet and coverslipped for light microscopic evaluation. The outlines of the SOC nuclei in light microscopic images were determined using the Nissl stain.

Those sections selected for EM analysis were fixed in 0.5% osmium tetroxide for 30 min, rinsed, and dehydrated through a series of graded alcohols and propylene oxide. Sections were then placed in unaccelerated Epon-Araldite resin and then transferred into a fresh batch of unaccelerated resin overnight. The sections were then embedded and flat mounted in accelerated resin between Aklar sheets at 65°C. The region of the plastic-embedded sections containing the labeled portion of the neuron was cut out of the 70-μm section and mounted on the flattened face of a plastic beam capsule. Because the labeled portion of the cell that is of interest may be tens of microns below the surface, the 70-μm section was resectioned into 3-μm sections that were placed on a glass coverslip. Each of the 3-μm sections was inspected with a light microscope and the section containing the labeled portion of the cell was selected. This section was removed from the glass coverslip and remounted on a beam capsule. A camera lucida drawing of the section face including the location of the labeled cell part was made and 70–80 nm thin sections were then cut and mounted on coated nickel grids. These thin sections were then stained with uranyl acetate and lead citrate and examined using a Philips CM-120 electron microscope.

Measurements of dendritic, somatic and axon terminal features from electron micrographs were made using ImageJ software (NIH). To determine percentage of synaptic coverage of a cell body or a dendrite, the length of the surface of the labeled structure was first measured and then the length of apposition of synaptic terminals on the labeled structure was measured. The circumference of each synaptic terminal was also measured. To approximate the surface area of the dendritic trees the following steps were taken. For two mLNTB cells and one pvLNTB cell, the length of all dendrites was measured and dendritic diameter was measured at 20 random locations. The dendrite surface area was approximated as a cylinder using the length of the total dendritic tree and the measured diameters. The cell body of one cell resembled a sphere while the other two resembled an ellipsoid so diameters were measured and the surface areas calculated for a sphere or an ellipsoid.

## Results

**FIGURE 1:**
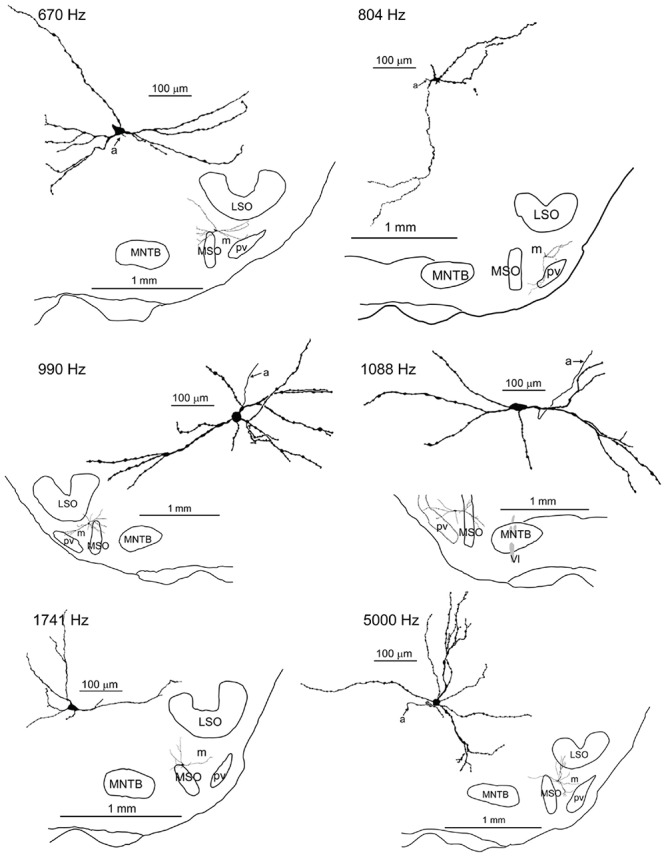
**Camera lucida drawings of six labeled neurons in the mLNTB.** The CF is indicated for each neuron. Insets show the location of the labeled unit on a coronal brainstem section. a, axon.

We obtained *in vivo* whole-cell recordings from 10 anatomically identified neurons in the brainstem area between LSO and MSO. A number of recordings, resulting in three labeled neurons, were located in a ventral, superficial location which we will argue below to be pvLNTB. Seven neurons were recorded more dorsally and revealed remarkable and unexpected morphological and physiological features. **Figure 1** shows camera lucida drawings of six of these neurons to illustrate their dendritic morphology and location in coronal brainstem sections. The extent of the dendritic trees was strikingly large. Most neurons were close to the lateral edge of the dorsal pole of the MSO, and had dendrites that traversed this nucleus. Their CFs were biased toward low frequencies. The similarity in morphology between neurons suggests that they constitute a hitherto unrecognized cell class in this area. We surmise that the neurons identified here are contained in a diffuse nucleus homologous to cat mLNTB. In the following, we first define LNTB subdivisions, then describe the morphology of the labeled cells at the light and EM level, and conclude with their physiology.

### LNTB Subdivisions

**FIGURE 2:**
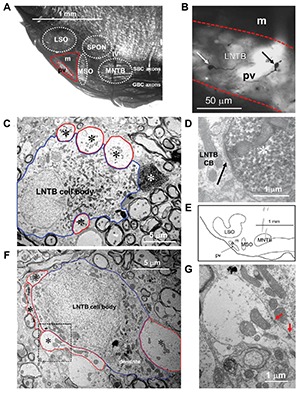
**Macroscopic and microscopic identification of pvLNTB. (A)** Plastic-embedded coronal section of gerbil brainstem showing the location of the main nuclei (white dotted outlines) embedded within the large bundle of axons known as the TB arising primarily from globular and spherical bushy cells in the cochlear nucleus. The area lateral to the MSO and ventral to the LSO (red solid line) is designated the LNTB. The area enclosed by the dotted red line is referred to as pvLNTB while the remaining dorsal, darker area is mLNTB. **(B)** Swellings (arrows) of labeled GBC axons within the pvLNTB (pv; red dotted lines). m: mLNTB **(C)** Electron micrograph of one of the labeled swellings (white asterisk) shown in B confirming that it is a synaptic terminal synapsing on a cell body in pvLNTB (blue outline) adjacent to other large unlabeled terminals (red outlines, black asterisks). **(D)** Synaptic specialization (arrow) of the labeled terminal in C. **(E)** Location of one of our physiologically characterized, labeled neurons (asterisk) in pvLNTB (outline). **(F)** Electron micrograph of the labeled pvLNTB cell body (blue outline) whose location is shown in E. Red outlines and asterisks show the large unlabeled synaptic terminals ending on this cell. **(G)** Enlarged view of the area outlined by a black dotted line in F showing synaptic specializations (red arrows).

When observed at the light microscopic level in plastic embedded sections (**Figure 2A**), the area lateral to the MSO and ventral to the LSO (solid red outline) in the gerbil has two distinct regions. In these sections darker areas represent locations where myelinated axons predominate and lighter areas where cell bodies predominate. As illustrated in **Figure 2A** there is an area in this region that is significantly lighter (red dotted outline). We hypothesized that this lighter area represents the pvLNTB while the more dorsal darker area, bordered by pvLNTB, LSO, and MSO, represents the mLNTB as defined in the cat (Spirou and Berrebi, 1996). Unlike the cat, the LSO of the gerbil does not have a ventral hilus so there can be no “hilar” LNTB region (hLNTB) in the gerbil.

One of the distinguishing features of cells in cat pvLNTB is the input to their cell bodies. Axons of GBCs in the cochlear nucleus on their way to form calyces of Held in the contralateral MNTB give off collaterals to the ipsilateral pvLNTB where they form large synaptic terminals on pvLNTB cells (Tolbert et al., 1982; Spirou et al., 1990; Smith et al., 1991). To determine whether cells in the area we have designated pvLNTB in gerbil have a similar input as in cat, we labeled a number of GBC axons. A small gross injection of biocytin was made in the ipsilateral TB at its ventral-most edge just below the MNTB where GBC axons are known to run (Smith et al., 1991). To verify that we had only labeled GBC axons, we searched for retrogradely labeled cells and anterogradely labeled axon terminals in the auditory brainstem. Virtually all cells backfilled by this injection were GBCs located in the nerve root area and virtually all the axons crossed the midline and ended as a calyx of Held in the contralateral MNTB (not shown). Almost no labeled terminals were seen in the MSO bilaterally – such terminals being a hallmark of spherical and not of GBC axons. At the light microscopic level many labeled axons and terminals were seen in the area designated pvLNTB. Many of these axonal swellings were large (**Figure 2B**, arrows). They were verified at the EM level to be synaptic terminals on cell bodies in the vicinity of other large unlabeled terminals synapsing on the same cell body (**Figures 2C,D**). They resemble the large PEP-19 immunoreactive terminals described in the cat pvLNTB (Berrebi and Spirou, 1998; Spirou et al., 1998), which supports our designation of this area in the gerbil as pvLNTB. **Figures 2E–G** illustrate a pvLNTB neuron cell body labeled via intracellular patch recording that receives large presynaptic terminals on its cell body. Only a few labeled collaterals of GBC axons were seen in the area of LNTB designated mLNTB. Terminal swellings on these axons were typically small.

Taken together, the homology of the features listed to cat pvLNTB make a convincing case for our designation of pvLNTB in the gerbil. The designation of the more dorsal, myelinated area as mLNTB (**Figure 2A**) is more tentative at this point, but is corroborated by several features described in the next section.

### Single LNTB Cells Labeled in Patch Electrode Experiments

#### mLNTB

**FIGURE 3:**
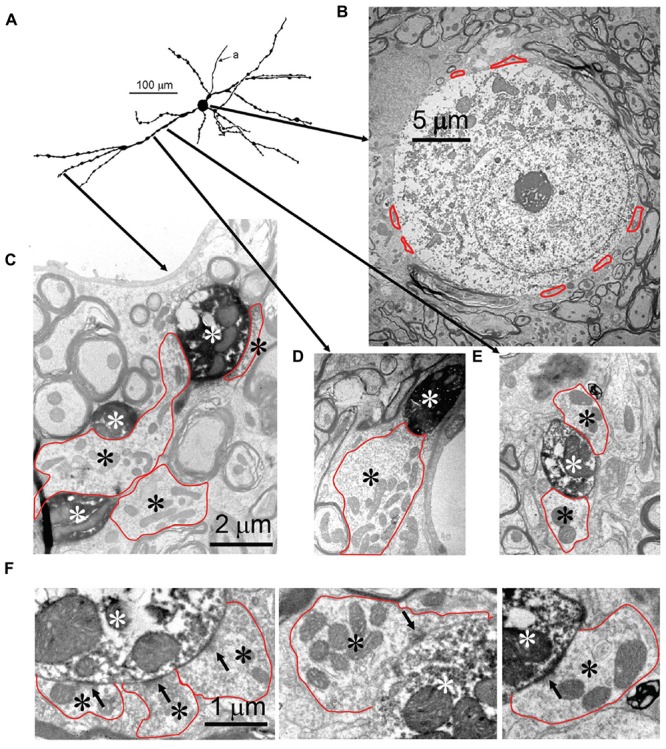
**Synaptic inputs to an mLNTB cell. (A)** Camera lucida drawing of the mLNTB cell. CF = 990 Hz. For its location in the SOC: see **Figure 1**. **(B)** Cell body. Red dotted outlines indicate synaptic terminals. **(C–E)** Electron micrographs illustrate the presence of large synaptic terminals (black asterisks, red outlines) on dendrites (white asterisks) at the location of the arrow origins shown in **(A)**. **(F)** Examples of synaptic specializations (arrows) at higher magnification seen in **(C–F)** or in adjacent sections.

Seven neurons were located dorsal to the pvLNTB, in the area we designate as mLNTB (**Figures 1** and **2A**; **Table 1**). The dendritic trees of these neurons extend over a large area, often covering much of LNTB and frequently running through the MSO. In most of the labeled LNTB neurons we were unfortunately unable to follow the axon to a point where we could, with confidence, verify their termination points. In two mLNTB cells, axons were sufficiently labeled to follow projections to the ipsilateral IC. EM of the cell bodies of these two mLNTB neurons showed that, in contrast to pvLNTB cells, the innervation of the cell body was very sparse with no large and few small terminals (**Figures 3A,B**). Instead, innervation of mLNTB cells appeared to reside primarily on the dendritic tree (**Figures 3C–F**). Note that an absence of large somatic terminals is also a property of cat hLNTB and mLNTB neurons (Spirou and Berrebi, 1996; Spirou et al., 1998). The cells showed beading of the dendritic trees (**Figure 1**), which we frequently see in cells retrieved from *in vivo* patch clamp recordings and which is probably an artifact. Electron microscopic analysis of these swellings did not reveal structural abnormalities of the surrounding tissue (data not shown). Dendritic beading is also regularly seen in patched cells retrieved from slices (Cao et al., 2007).

**Table 1:** Overview of LNTB cell properties.

Cell number	Intra/Extra	Subdivision	CF (Hz)	Ih sag	AP	Short tones responses	ITD sensitive	Terminal area coverage	Projects to		Figures
									SPON	CN	
m1	I	mLNTB	670		C	Chop					1, 6D, 7A, 9A, 10A, 11E
m2	I	mLNTB	804		I	Reg					1, 7B, 9B, 10B
m3	I	mLNTB	990	+	B	O	+	17.4%	+		1, 3, 4A, 7C, 9C, 10C, 11B, 13D, 14A–D
m4	I	mLNTB	1088		B	O	+	26.3%	+		1, 9D, 10D, 11A, 13A/C, 14E
m5	I	mLNTB	1741		-	-					1, 9E, 10E, 13B/E
m6	I	mLNTB	5000	+	B	Chop	+				1, 7D, 9F, 10F, 11D, 14F
m7	I	mLNTB				-					6C, 11C
p1	I	pvLNTB	1060	+	I	PL_N_		38.4%			2F, 6A, 7F, 8B, 12B/D
p2	I	pvLNTB	1650		I	PL_N_					6B, 8A, 12A/C
p3	E	pvLNTB	1979		I	PL_N_					12E
p4	I	pvLNTB	∼900–1300		B	PL		25.4%		+^∗^	5, 7E, 8C, 12F

*In the column Intra/Extra, “I” indicates intra- and “E” indicates extracellular recording. CF is indicated when known. Ih sag: presence of Ih sag in response to hyperpolarizing current injections. AP: indicates whether action potentials were obtained in response to contralateral (C), ipsilateral (I) or ipsilateral and contralateral (B) sound. PSTH: Chop, chopper; Reg, regular; O, onset; PL_N,_ primary-like-with-notch; PL, primary-like. Terminal area coverage: percentage of cell body covered with synaptic terminals. The column “Figures” lists the figure panels in which the neuron is illustrated. ^∗^: the axon of this cell projects toward but fades before reaching the CN.*

**FIGURE 4:**
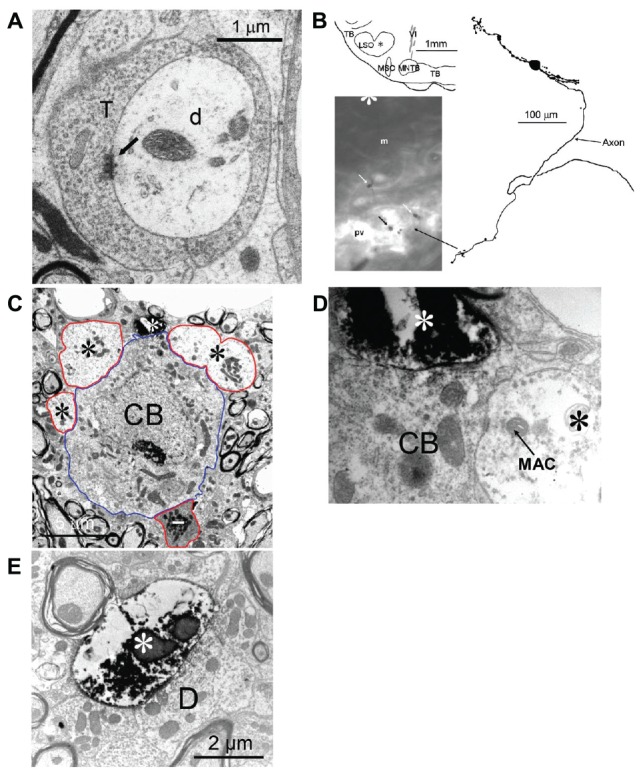
**Connections between the LNTB, SPON and LSO. (A)** Synaptic terminal of an mLNTB cell axon collateral on a dendrite in the SPON. Arrow indicates synaptic specialization. T: mLNTB synaptic terminal; d: SPON dendrite. **(B–E)** The LSO projects to the LNTB. **(B)** Efferents from a labeled LSO neuron form large endings in the pvLNTB. Top left: Location of the LSO cell body. Right: Camera lucida drawing of the LSO principal cell. The main axon initially headed ventrally then turned medially on its way to the ipsilateral IC. Bottom left: at the point where the main axon turns medially an axon collateral is given off that heads ventrally into the LNTB. The swellings seen toward the end of this collateral are indicated with arrows. **(C)** Electron micrograph of one of the swellings of the LSO axon collateral, shown in A, synapsing (white asterisk at 12 o’clock) on a pvLNTB cell body (blue outline) that is also contacted by several large presynaptic terminals (red outlines, black asterisks). Larger profiles of the LSO terminal seen in adjacent sections are not shown. Also contacting the cell body is what appears to be an inhibitory terminal with a darker axoplasm (red outline, white dash). **(D)** Enlarged electron micrograph of the same labeled LSO axon terminal (white asterisk) shown in **(C)** synapsing on a pvLNTB cell body (CB), immediately adjacent to a large unlabeled terminal (black asterisk) that contains a structure resembling a “mitochondrial-associated adherens complex” (MAC). MACs are also found in cat GBC axon terminals in pvLNTB (Spirou et al., 1998). **(E)** Electron micrograph of another LSO axon terminal (white asterisk) synapsing on a large dendrite **(D)** in pvLNTB.

The two mLNTB cells described above with axons projecting to IC both gave off an axon collateral that projected to the SPON. **Figure 4A** shows one of these terminals containing round vesicles and displaying an asymmetric synaptic specialization on a dendrite in SPON.

#### pvLNTB

**FIGURE 5:**
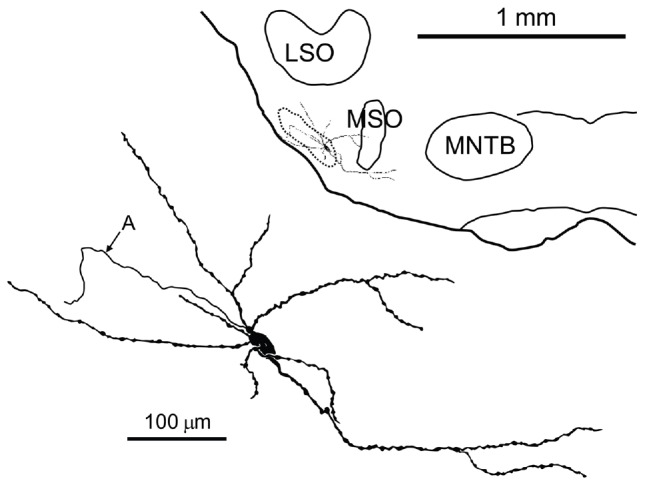
**Camera lucida drawing of a labeled neuron in the pvLNTB with an extensive dendritic tree.** Inset shows the location of the labeled cell on a coronal brainstem section. A, axon; LSO, lateral superior olive; MNTB, medial nucleus of the TB; MSO, medial superior olive.

Three of the 10 neurons labeled were located in the clear superficial area we have designated pvLNTB (**Figure 2A**, dotted red outline; **Table 1**). **Figure 2E** shows the location of one of these neurons (asterisk). The superficial location of these neurons hampers successful patch recording and in only one of the neurons was the labeling dark enough to make a camera lucida drawing (see below, **Figure 5**). We performed EM on two of these pvLNTB neurons. We observed several large terminals synapsing on the cell body (**Figures 2F,G**), which correspond to the large terminals seen on pvLNTB neurons in the cat (Stotler, 1953; Adams, 1983; Spirou and Berrebi, 1996; Spirou et al., 1998). Interestingly, large presynaptic endings have been shown to occur in the human LNTB as well (Kulesza, 2014). Spirou and Berrebi (1996) showed that arrays of large terminals are characteristic of the pvLNTB.

We also found a previously unreported input to LNTB. In a separate set of experiments we targeted LSO cells for intracellular recording, physiological characterization and labeling. **Figures 4B–E** illustrate a labeled LSO principal cell whose axon headed to the ipsilateral IC. While still in the SOC this axon gave off a collateral branch that projected to the LNTB (**Figure 4B**). This neuron had a CF of 12 kHz and was sensitive to ILD at CF (data not shown). Some of the swellings of these labeled axons in the pvLNTB were large (**Figure 4B**, arrows). At the EM level we confirmed on several sections that these large swellings could synapse on the cell bodies near other large unlabeled terminals (**Figures 4C,D**) or on large dendrites (**Figure 4E**). Some of these large unlabeled terminals contained mitochondrial-associated adherens complexes (MACs, **Figure 4D**). MACs are one of the identifying features of globular bushy axon terminals in pvLNTB cells in the cat, so their presence is consistent with the unlabeled terminals here being from GBCs (Berrebi and Spirou, 1998). Finally, we observed darker terminals with inhibitory features contacting pvLNTB cell bodies (**Figure 4C**, white dash), that may correspond to local terminals of LNTB neurons that have been described in gerbil slice (Roberts et al., 2014).

The extent of dendritic trees of the neurons in mLNTB was strikingly large when compared to the majority of LNTB neurons, labeled in the same species in a thick slice preparation (Roberts et al., 2014), that were shown to project to the MSO. These cells had small dendritic trees confined primarily to a small space surrounding the cell body, suggesting that the LNTB cells labeled by these authors are part of the pvLNTB. We did, however, record from and label one cell in pvLNTB that had a large dendritic tree similar to mLNTB cells (**Figure 5**). The dendrites of this cell extended a considerable distance into the mLNTB as well as into the MSO. The axon of this pvLNTB did not head medially toward the MSO (as in Roberts et al., 2014) but instead laterally into the TB on a course similar to that taken by axons of cat LNTB cells on their way to the cochlear nucleus (Spangler et al., 1987). As described below, this anatomically unusual pvLNTB cell also had physiology that differed from our other labeled pvLNTB cells.

#### Quantification: Cell Body

We quantitatively compared the synaptic inputs to the cell bodies of two labeled mLNTB cells whose axons were traced to the inferior colliculus and of four pvLNTB cells (one intracellularly labeled cell; one unlabeled cell that received labeled terminals from GBCs; one unlabeled cell that received labeled terminals from an LSO principal cell; and the unusual intracellularly labeled cell that projected toward the cochlear nucleus, but could not be traced to its termination there due to fading of the label; **Figure 5**). The two mLNTB cell bodies were sparsely innervated, with terminal coverage averages of 17.4 and 26.3% (length of terminal apposition), and the synaptic terminals were small (average terminal circumference = 5.27 μm and 6.16 μm; rarely (<7%) larger than 11 μm in circumference and never over 12 μm). In contrast three out of four pvLNTB cell bodies (with the exception of the one projecting toward the cochlear nucleus) had larger terminal coverage averages than the mLNTB cell bodies (length of synaptic apposition 38.4, 42.6, and 54.2%), and the synaptic terminals were larger than on mLNTB cell bodies (average circumference = 13.4 μm, 10.5 μm and 9.6 μm; 37.7% of the terminals were larger than 12 μm with some greater than 20 μm). For the cell with the labeled globular bushy terminals the labeled terminals were amongst those classified as the larger terminals. In contrast the terminal coverage of the pvLNTB cell body projecting toward the cochlear nucleus (**Figure 5**) differed from the other pvLNTB cells and more closely resembled the mLNTB cells in terms of terminal coverage average (length of apposition, 25.37%) and synaptic terminal size (average circumference, 6.95 μm, none over 12 μm).

#### Quantification: Dendrites

We also evaluated the terminal input to the dendritic tree of the two mLNTB cells projecting to the inferior colliculus. In both cases, when compared to the soma coverage of these same cells, a significantly larger portion of the dendritic tree surface was covered with synapses (53.8, 40.5%) even at the very end regions of a given dendrite. Our measurements also showed that the surface area of the dendritic trees of these two cells was over 20 times the surface area of the cell bodies. Taken together these features indicate that the vast majority of the input to these cells is located along the length of the dendritic trees. The average size of the dendritic terminals (6.83, 6.3 μm) was similar to those seen on the cell bodies but unlike the cell body synapses, some of these dendritic terminals were larger than 12 μm. An evaluation of the dendritic tree of the unusual pvLNTB cell (**Figure 5**) showed that again this cell more closely resembled the mLNTB cells in that 51.2% of its dendritic tree was covered with synapses and the dendritic surface area was over 20 times larger than that of the somata both of which indicate that the vast majority of its input is on the dendrites.

Overall, these findings are consistent with the proposal that the LNTB of the gerbil consists of (at least) two subdivisions, pvLNTB and mLNTB, with most but not all cells in the two regions displaying characteristic anatomical features.

### Physiology

#### Spontaneous Activity

**FIGURE 6:**
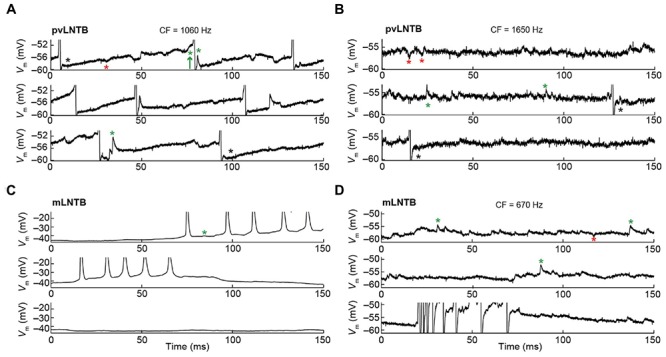
**Spontaneous activity of LNTB neurons.** CF is indicated when known. **(A)** Spontaneous activity in a pvLNTB neuron. Black asterisks: spike afterhyperpolarization shows double undershoot, as described in gerbil slice (Roberts et al., 2014). Red asterisks: IPSPs. Green asterisks: EPSPs. Green arrow: EPSP that results in an action potential. **(B)** Spontaneous activity in another pvLNTB neuron. Symbols as in **(A)**. **(C,D)** Spontaneous activity in two mLNTB neurons. Symbols as in **(A)**.

The membrane potential (*V*_m_) data obtained during intracellular recordings in the absence of sound was characterized by many spontaneous events including synaptic events and, in several neurons, spikes (**Figure 6**). In pvLNTB as well as in mLNTB neurons, we observed spontaneous EPSPs (**Figures 6A–D**, green asterisks). Sometimes a spontaneous EPSP could be seen giving rise to an action potential (**Figure 6A**, green arrow) but there were also a large number of subthreshold events in cells from both regions. A number of spontaneous IPSPs were identified (**Figures 6A,B,D**, red asterisks). Interestingly, action potentials were followed by a biphasic afterhyperpolarization in the pvLNTB (**Figures 6A,B**, black asterisks), similar to spike afterhyperpolarizations reported from *in vitro* recordings from gerbil LNTB neurons which were referred to as “double undershoot” (Roberts et al., 2014). Bursts of spontaneous action potentials were frequent in one mLNTB neuron, and occasionally observed in three additional mLNTB neurons (out of seven neurons; as in **Figures 6C,D**), and in one out of three pvLNTB neurons.

#### Responses to Current Injections

**FIGURE 7:**
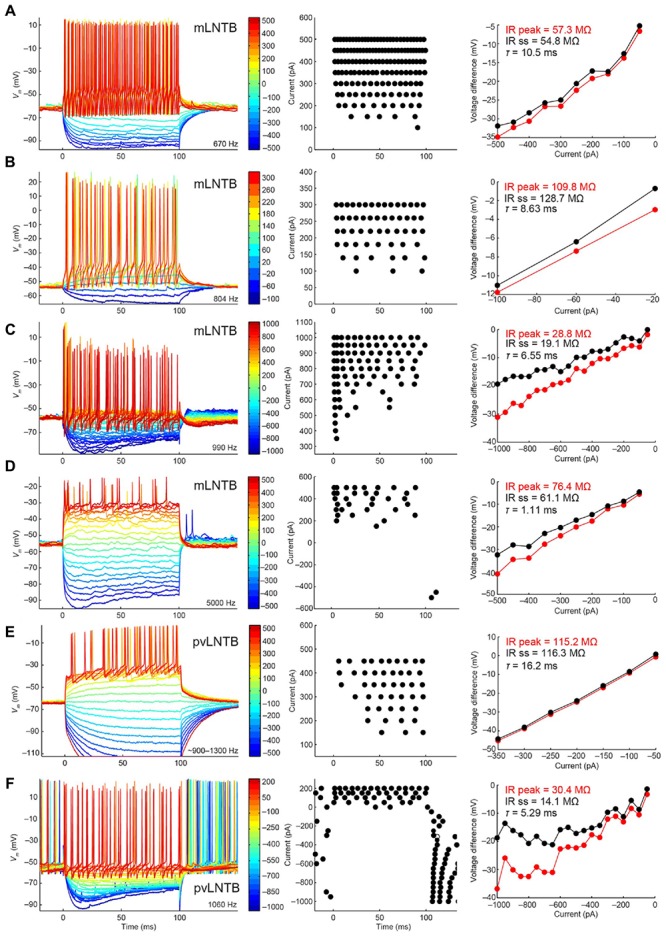
**Voltage response to current steps for LNTB neurons.** Voltage response to current injections is shown for six neurons: four mLNTB neurons **(A–D)** and two pvLNTB neurons **(E,F)**. Left panels show voltage responses to hyperpolarizing and depolarizing current steps (color legend shows current amplitude in pA). Middle panels are dot rasters showing the action potentials from the left panels. Right panels are IV plots of the hyperpolarizing responses from the left panels, plotted separately for the peak and steady state response. The peak and steady state input resistance (IR) as well as the membrane time constant (*τ*) are mentioned in each panel. The bridge was sometimes not well-balanced, especially for the cell shown in **(E)**. A *post hoc* calculation of the resistance showed that the true series resistance for this cell is about 108.6 MΩ (the bridge was balanced for 75.7 MΩ). The input resistance mentioned in **(E)** was corrected for this value.

We show the voltage response to current step injections for six neurons in **Figure 7**. **Figures 7A–D** shows mLNTB neuron responses, **Figures 7E,F** pvLNTB neuron responses. Interestingly, all neurons responded repetitively with multiple spikes to depolarizing steps (**Figure 7**, left and middle panels), as was seen *in vitro* (Roberts et al., 2014). This is in contrast to the single action potential that is typically generated by MSO neurons at the onset of such depolarizing steps (Scott et al., 2005), or the similar onset spike response seen in MNTB (Roberts et al., 2014) or cochlear nucleus bushy neurons (Oertel, 1983; Manis and Marx, 1991; Trussell, 1999). When hyperpolarizing steps were employed, an initial “sag” was seen in some mLNTB (**Figures 7C,D**) as well as in some pvLNTB neurons (**Figure 7F**). This is also demonstrated by a larger peak than steady-state input resistance in these units (**Figures 7C,D,F**, right panels). This sag is characteristic of the presence of an *I*_h_ current (Trussell, 1999; Roberts et al., 2014). We did not find a systematic difference between mLNTB and pvLNTB neurons in terms of spiking pattern, input resistance or membrane time constants (**Figure 7**, right panels). Notably, for mLNTB cells, membrane time constants decreased with increasing CF (indicated in panels in left column).

#### Responses to Monaural Tones – pvLNTB

**FIGURE 8:**
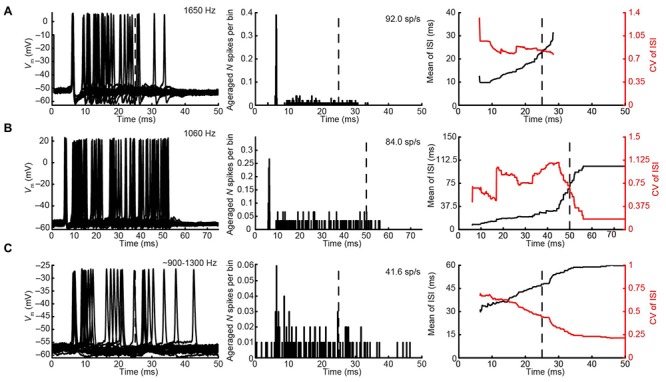
**Ipsilateral responses to short tones at CF for 3 pvLNTB neurons. (A–C)** Each row corresponds to one pvLNTB neuron. Left panels: stacked intracellular responses to 20 repetitions of short monaural ipsilateral tones at CF. CF is indicated when known; for the neuron in C a frequency tuning function was not available but a CF-range is estimated from the response latency to short tones. For this neuron responses to 900 Hz tones are shown. Middle panels: PSTH. Bin size = 0.1 ms. Spike rate is indicated. Right panels: mean (black line) and CV (red line) of ISIs. The neuron in C corresponds to that in **Figure 5**. Stimulus SPL and number of repetitions for the PSTH (middle column) were; **(A)** 80 dB SPL/100 repetitions, **(B)** 75 dB SPL/30 repetitions, **(C)** 90 dB SPL/100 repetitions.

The CF was obtained for three pvLNTB neurons (1060, 1650, and 1979 Hz). Three out of four pvLNTB neurons responded only to monaural ipsilateral (and not to monaural contralateral) tones, and displayed spike patterns to short tones at CF that could be classified as primary-like-with-notch. One of these four pvLNTB cells was recorded extracellularly, displayed a similar firing pattern (monaural ipsilateral and primary-like-with-notch), and corresponded to pvLNTB in terms of recording depth (data not shown). These primary-like-with-notch responses were characterized by a well-timed first spike that occurred around 5–7 ms after the stimulus onset for high sound levels, followed by an absence of spiking for about 2.5 ms (the notch), then a low level of sustained activity (**Figures 8A,B**). This primary-like-with-notch response is reminiscent of the PSTH of GBCs in the cochlear nucleus that provide a major excitatory input to these pvLNTB cells, although in GBCs the notch is shorter than 2 ms (Smith et al., 1991). Sensitivity to ITD or ILD was not systematically tested in these four pvLNTB neurons, as recording time was limited. For the two pvLNTB neurons in which ITD-sensitivity was tested with a binaural beat, the result was negative. A regularity analysis of ISIs (**Figures 8A,B**, right panels) shows that the mean ISI increases over time, and that the CV of ISIs is generally above 0.5 for these responses, which has been observed for primary-like and primary-like-with-notch responses in the cat ventral cochlear nucleus (Young et al., 1988).

Unlike the other pvLNTB neurons, the pvLNTB neuron with the extended dendritic tree (**Figure 5**) had a high spontaneous spike rate, was binaural – responding with an excitatory response to sound from either ear, and had an ipsilateral PSTH resembling that seen in auditory nerve fibers that is typically called primary-like rather than primary-like-with-notch (**Figure 8C**). This neuron was not sensitive to ITD, and its CV did not reach values as high as the pvLNTB neurons with the primary-like-with-notch responses (**Figure 8C** vs. **Figures 8A,B**).

#### Responses to Monaural Tones – mLNTB

**FIGURE 9:**
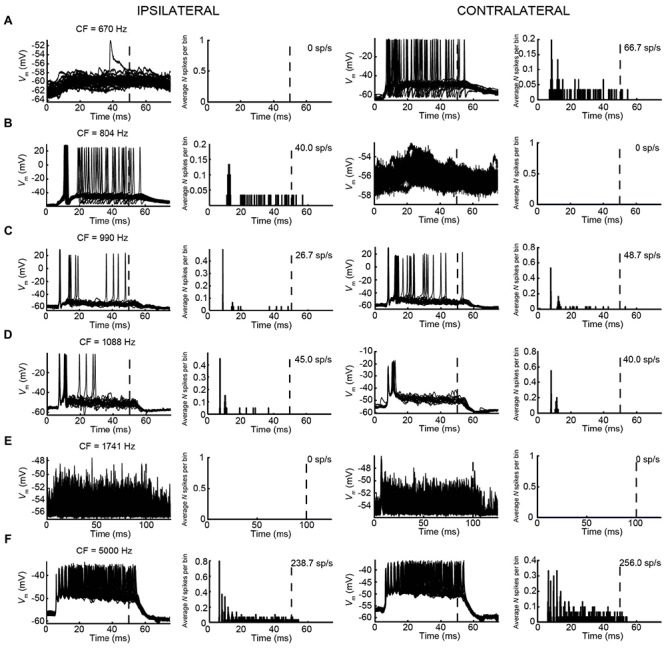
**Responses of mLNTB neurons to short tones at CF.** *V*_m_ (left) and PSTH (right) of responses to ipsilateral (two leftmost columns) and contralateral (two rightmost columns) short tones at CF for 6 mLNTB neurons. Bin size = 0.1 ms. Spike rate is indicated for each response. **(A)** 80 dB SPL/30 repetitions; **(B)** 85 dB SPL/30 repetitions; **(C)** 80 dB SPL/30 repetitions; **(D)** 70 dB SPL/20 repetitions; **(E)** 70 dB SPL/50 repetitions; **(F)** 70 dB SPL/30 repetitions.

We obtained the CF for six mLNTB neurons, which ranged from 670 to 5000 Hz. The response of mLNTB neurons to short tones at CF is shown in **Figure 9**. To ipsilateral sound, 4 out of 6 neurons responded with spikes (**Figures 9B–D,F**). The remaining two neurons showed an increase in depolarizing subthreshold activity (**Figures 9A,E**). Four out of six neurons responded with spikes to contralateral short tones (**Figures 9A,C,D,F**), but these were not the same subgroup as the cells responding to ipsilateral tones. One neuron showed, in response to CF tones (**Figure 9E**), an absence of spiking but fast EPSPs as are observed in MSO (Franken et al., 2015), and infrequent onset spikes in response to short contralateral tones in the tuning curve tail (data not shown). This neuron was located very close to the outline of the MSO (**Figure 1**, CF = 1741 Hz). The fact that half of the neurons could be stimulated from either ear fits with the extended dendritic trees of these neurons, which reached both lateral as well as medial to the MSO where respectively, ipsilateral and contralateral excitatory inputs to MSO are abundant (**Figure 1**).

One striking difference between pvLNTB and mLNTB responses could be seen during sound presentation. During ipsilateral sound the membrane potential from mLNTB cells (**Figure 9**), but not pvLNTB cells (**Figure 8**), showed a sustained subthreshold depolarization, suggesting either a larger number of subthreshold inputs and/or a slower membrane time constant. Because the latter was not found in the analysis of responses to hyperpolarizing current steps (**Figure 7**, right panels), and in view of the more extended dendritic trees of mLNTB neurons, it is plausible that the former factor is more important.

**FIGURE 10:**
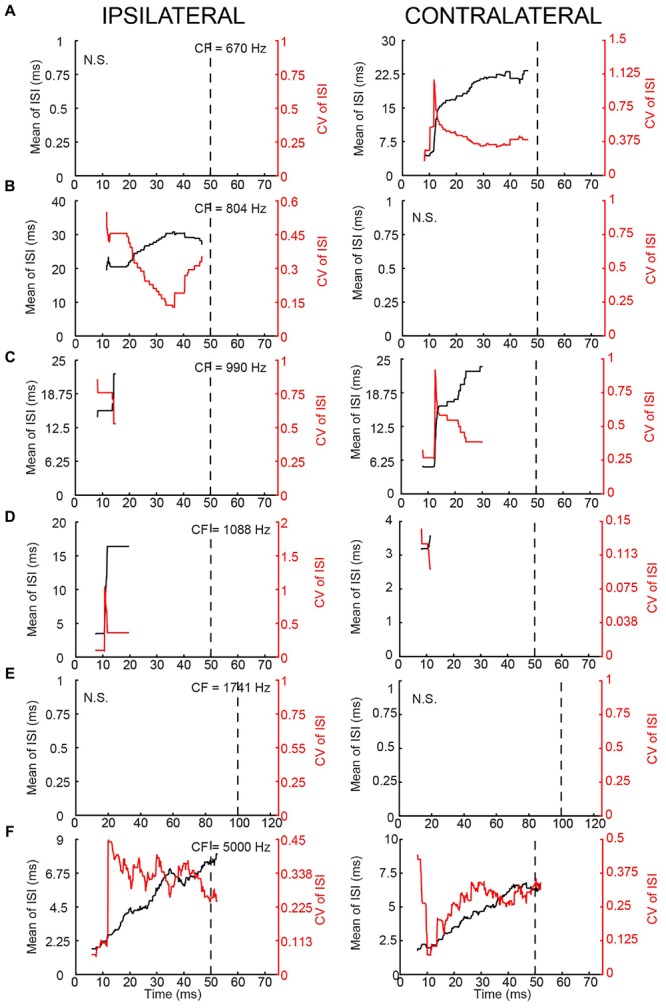
**Regularity analysis of mLNTB responses to short tones at CF. (A–F)** Mean (black line) and CV (red line) of ISIs of responses to monaural tones at CF, for the corresponding neurons in **Figure 9**. N.S., no spikes.

In terms of PSTH classification, the spike responses of mLNTB cells were diverse. When there was a sustained response (**Figures 9A,B,F**), the PSTH was chopper (**Figure 9F**) and/or the response tended to be regular, as shown by a CV of ISIs <0.5 over most of the response duration (**Figures 10A,B,F**), using the criterion reported for chopper units in the ventral cochlear nucleus (Young et al., 1988). Two neurons displayed mainly onset responses to stimulation of either ear (**Figures 9C,D**). However, the PSTH had a tendency toward being multimodal with an early secondary peak. This corresponds to a low CV early on for the ISI, which increases to >0.5 afterward (**Figures 10C,D**).

**FIGURE 11:**
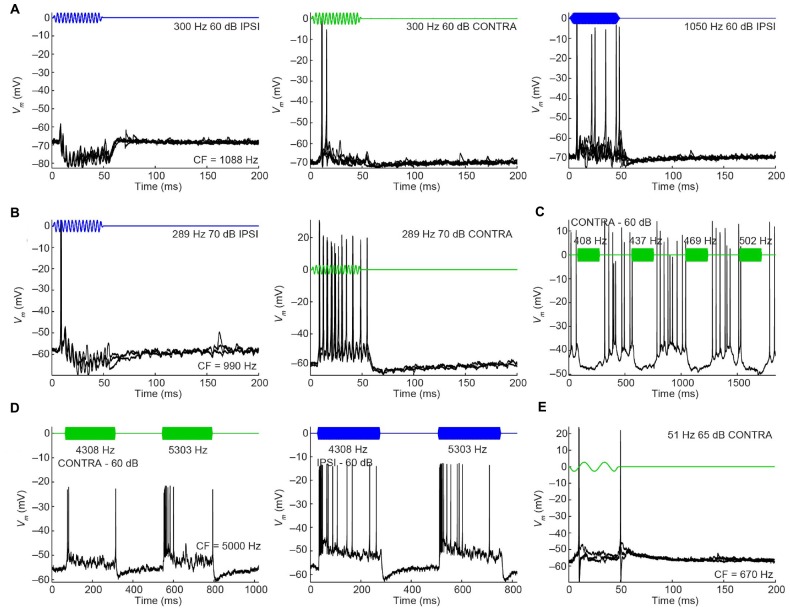
**Inhibitory features in responses to sound of mLNTB neurons. (A–E)** Stacked responses to monaural stimuli for 5 mLNTB neurons. Stimuli are shown in blue (ipsilateral stimulation) or green (contralateral stimulation). Number of repetitions shown: **(A)** 5; **(B)** 3; **(C)** 1; **(D)** 1; **(E)** 3.

Another difference between mLNTB and pvLNTB neurons concerns the presence of inhibition. In several mLNTB neurons we observed features during sound stimulation suggesting inhibition. **Figure 11** illustrates responses of 5 neurons. In two neurons (**Figures 11A,B**), responses to low-frequency ipsilateral tones contained clear IPSPs, as opposed to the responses to contralateral tones or higher frequency ipsilateral tones. Another mLNTB neuron (**Figure 11C**) displayed inhibitory responses to tones throughout the frequency spectrum, especially to contralateral tones, and, somewhat less clear or at higher levels, also to ipsilateral tones (not shown). In two other mLNTB neurons (**Figures 11D,E**), spiking at stimulus offset was observed to contralateral tones, which might correspond to release from inhibition. Clear signs of inhibition in response to sound were not observed in pvLNTB neurons.

#### Phase-Locking and Sensitivity to ITD

**FIGURE 12:**
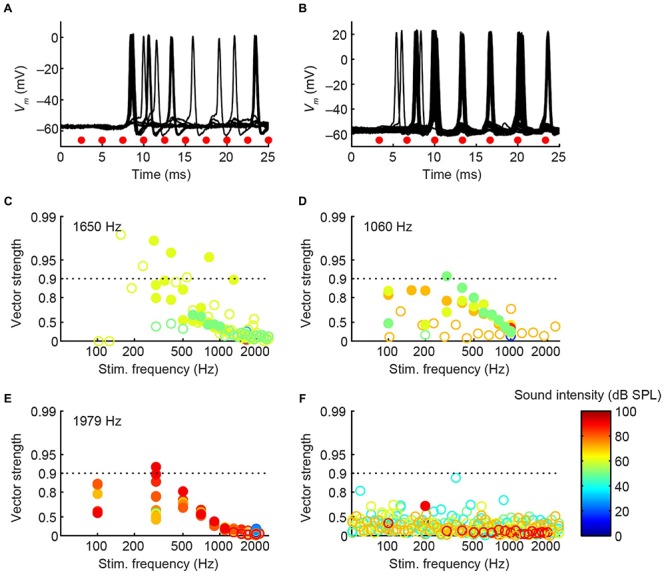
**Phase-locking of pvLNTB neurons to tones. (A)** Stacked responses of a pvLNTB neuron to 400 Hz tones at 60 dB SPL (CF = 1650 Hz). Red dots indicate stimulus periods. VS is 0.78 (α ≤ 0.001). **(B)** Responses of another pvLNTB neuron (CF = 1060 Hz) to 300 Hz tones at 50 dB SPL. VS is 0.91 (α ≤ 0.001). **(C–F)** VS as a function of stimulus frequency for four pvLNTB neurons. **(C)** Corresponds to the neuron in **(A,D)** corresponds to the neuron in **(B)**. Filled symbols correspond to data with significant phase-locking. Colors indicate sound levels (scale in **F**). CF, if known, is indicated in the panel. Note that the ordinate uses an expansive, approximately isovariance, scale, where (1-VS) is plotted on an inverted log axis (Johnson, 1980; Joris et al., 1994a). Dashed horizontal line indicates a VS of 0.9.

In cat, the LNTB is thought to provide input to the ipsilateral MSO (Cant and Hyson, 1992), and pvLNTB neurons are reported to be glycinergic (Spirou and Berrebi, 1997), so the neurons in this subdivision are probably providing ipsilateral inhibition to the MSO. A phase-locked glycinergic LNTB input has been proposed to perform a critical role in ITD tuning (Myoga et al., 2014). Although recent *in vivo* intracellular MSO recordings with pharmacological manipulations do not support this claim (Franken et al., 2015), it remains a critical issue to examine whether these inhibitory LNTB neurons could provide precise timing information, which at present is unclear. Although none of the pvLNTB neurons of our limited example was tuned to the very low frequencies at which ITD-sensitivity is most marked, we studied these neurons with tones below CF. Example response traces show that pvLNTB neurons can generate phase-locked action potentials (**Figures 12A,B**). We calculated the VS (Goldberg and Brown, 1969) of action potentials for pvLNTB responses to ipsilateral tones (**Figures 12C–F**): three pvLNTB neurons showed significant phase-locking of suprathreshold activity to a range of stimulus frequencies. In the unusual pvLNTB neuron with the extended dendritic tree, VS was significant at only one frequency/SPL combination (**Figure 12F**).

**FIGURE 13:**
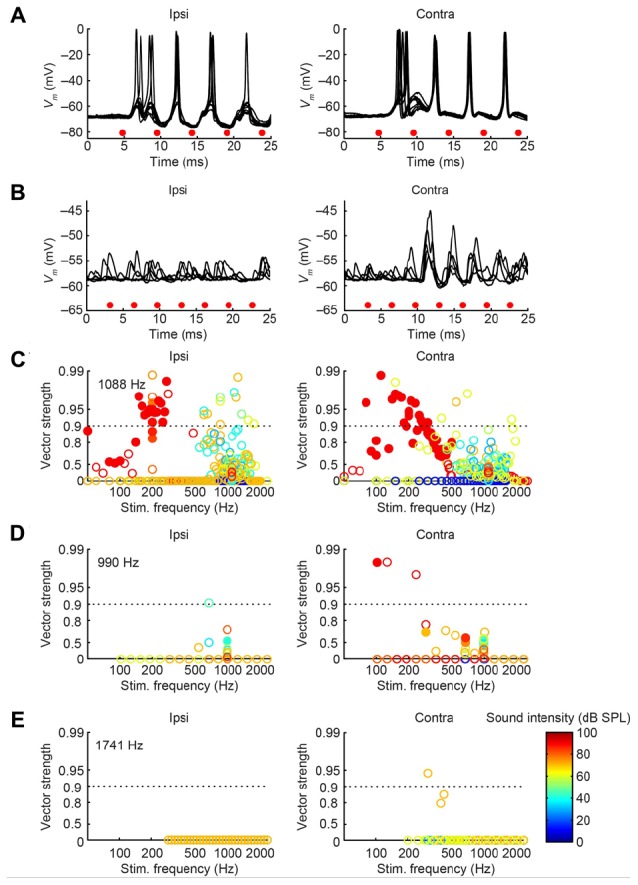
**Phase-locking of mLNTB neurons to tones.** Left column: responses to ipsilateral tones. Right column: responses to contralateral tones. **(A,B)** *V*_m_ traces for two neurons; **(C–E)** plots of VS for three neurons. **(A)** Stacked responses to ipsilateral (left) and contralateral (right) tones of 210 Hz at 90 dB SPL (CF = 1088 Hz). Red dots indicate stimulus periods. VS is 0.94 (α ≤ 0.001) for ipsilateral stimulation and 0.96 (α ≤ 0.001) for contralateral stimulation. **(B)** Similar to **(A)**, for another neuron (CF = 1741 Hz), for tones of 309 Hz at 70 dB SPL. **(C)** VS as a function of stimulus frequency, for the neuron in **(A)**. Filled symbols correspond to data with significant phase-locking. Colors indicate sound levels (scale in **E**). CF is indicated in the left panel. Dashed line indicates a VS of 0.9. **(D,E)** Similar to **(C)**, for other mLNTB neurons. The neuron in **(E)** corresponds to the neuron in **(B)**.

Perhaps more surprisingly, 3/7 mLNTB neurons also showed significant phase-locking of spikes to ipsilateral (**Figure 13**, left column) and/or contralateral (right column) stimulation. **Figures 13A,B** shows voltage traces for two neurons. The spiking responses in **Figure 13A** show “high-sync” phase-locking with VS values >0.9 (see figure legend). Moreover, in response to contralateral stimulation, this enhanced phase-locking is accompanied by entrainment, i.e., a precisely timed spike is fired on every stimulus cycle. VS values for this neuron are shown over a range of frequencies and SPLs in **Figure 13C**. In a second neuron (**Figure 13B**), there was little spiking but a subthreshold phase-locked response is clearly present to contralateral stimulation and only weakly to ipsilateral stimulation. VS values for this neuron are shown in **Figure 13E**. In 3 out of 5 remaining mLNTB neurons, VS values did not reach significance due to a small number of stimulus repetitions or a small number of spikes.

**FIGURE 14:**
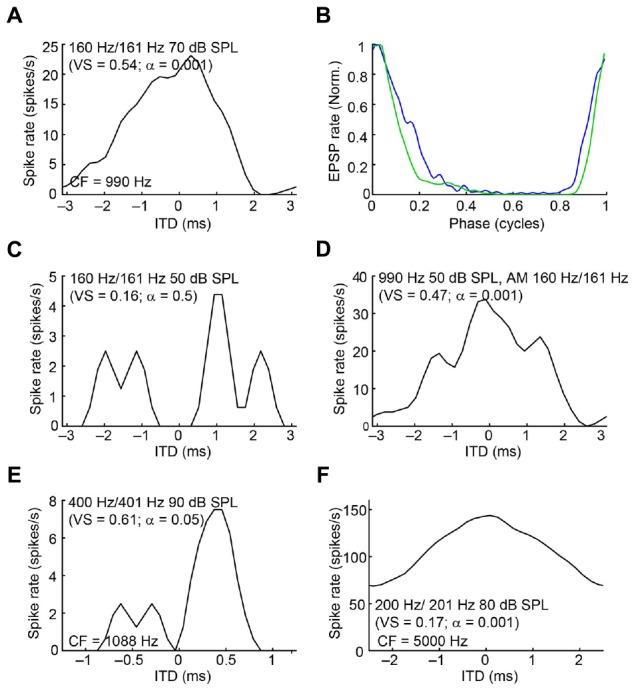
**ITD tuning of mLNTB neurons. (A)** ITD function of an mLNTB neuron in response to a binaural beat stimulus (160 Hz/161 Hz, 70 dB SPL) **(B)** Cycle histograms of subthreshold EPSPs from the neuron in A in response to monaural tones corresponding to the binaural beat stimulus used in A (green: response to contralateral stimulus, blue: response to ipsilateral stimulus). **(C)** ITD function of the neuron in A to the same binaural beat stimulus as in A but at a lower SPL (50 dB SPL). **(D)** ITD function of the neuron in **(A,C)** to an amplitude-modulated tone with a binaurally beating envelope. Carrier frequency: 990 Hz. Modulation frequency: 160 Hz/161 Hz. Sound intensity: 50 dB SPL. **(E,F)** Similar to **(A)**, for 2 other mLNTB neurons. Respective stimuli: 400 Hz/401 Hz, 90 dB SPL; 200 Hz/201 Hz, 80 dB SPL; CF, vector strength (VS) and Rayleigh α of ITD tuning are indicated in **(A,C–F)**.

Because mLNTB neurons are often binaural and can display phase-locking to monaural stimuli, we examined whether they have ITD tuning. Three out of 5 mLNTB neurons tested indeed showed sensitivity to ITD in response to binaural beats presented at low frequencies (**Figures 14A,E,F**; the CFs are stated above the abscissa): response rate varied as a function of the instantaneous ITD in the stimulus. Interestingly, these three neurons responded with action potentials to tones at CF to either ear (**Figures 9C,D,F**). When the neuron illustrated in **Figure 14A** was tested to the same stimulus (160/161 Hz) at a lower SPL (50 dB SPL), there was no significant ITD-sensitivity (**Figure 14C**), but if a CF tone at that intensity was sinusoidally amplitude-modulated at 160/161 Hz, ITD-sensitivity to the stimulus envelope was present (**Figure 14D**). For both types of stimuli, the ITD curves where broadly centered near 0 ITD (**Figures 14A,D**), consistent with the phase of monaural subthreshold events (**Figure 14B**). Indeed, when the frequencies composing the binaural beat of **Figure 14A** were played monaurally, the EPSPs recorded in this neuron were phase-locked with similar phase to the contralateral tone (**Figure 14B**, green line; best phase *BP_c_* = 0.051 cycles) and the ipsilateral tone (**Figure 14B**, blue line; best phase *BP_i_* = 0.054 cycles). This type of behavior suggests that this mLNTB neuron may be a primary coincidence detector, rather than inheriting its ITD-sensitivity from MSO neurons.

## Discussion

We performed intracellular recording and labeling of neurons in gerbil, in a brainstem area bound by the two main binaural nuclei of the SOC: the MSO and LSO. Our most surprising finding in this rather sparsely populated region is that neurons with large dendritic trees are found with striking and unexpected morphological and physiological properties. The majority of these neurons were recorded at a fairly dorsal location in the area between MSO and LSO and showed very large dendritic trees that even traversed the nuclear boundaries of the MSO. Moreover, these neurons were often binaural and could show ITD-sensitivity and highly phase-locked (“high-sync”) firing to low frequencies. We surmise that these neurons are part of the mLNTB, as defined in cat (Spirou and Berrebi, 1996), based on their general location and the observation that they receive most of the synaptic input on their dendrites. We also recorded from a smaller sample of neurons located in an area that we define as pvLNTB, based on multiple features that are found in common with the cat (Spirou and Berrebi, 1996). pvLNTB is characterized by neurons receiving large somatic excitatory synapses from GBCs, but also inhibitory inputs – many of which are thought to arise from axon collaterals of other (pv)LNTB neurons (Roberts et al., 2014). We found that these neurons can also get input from LSO. The neurons displayed primary-like-with-notch responses and their phase-locking capability could sometimes be classified as “high-sync,” at least in the tuning curve tails.

Although our sample is small, these are the first *in vivo* intracellular recordings from identified LNTB neurons. To some extent, our findings are in line with properties expected from previous anatomical studies and extracellular recordings (two basic cell types differing in somatic innervation pattern; diverse responses to pure tones including phase-locked responses and responses reminiscent of those of GBCs). On the other hand, the extent of dendritic branching in mLNTB neurons and the presence of “high-sync” responses and ITD-sensitivity were not anticipated.

### LNTB Inputs

We show that, as in the cat, one of the major excitatory inputs to LNTB is from ipsilateral GBCs that terminate primarily on pvLNTB cell bodies as large terminals. No large somatic terminals are seen on mLNTB cells. mLNTB neurons respond robustly to auditory stimuli so it would seem that such responses would require more than the sparse excitatory inputs that we have noted from GBC axons. What might the origin of such inputs be? Doucet and Ryugo (2003, 2006) reported that, in rat and cat, multipolar cells designated planar or T-stellates in the ventral cochlear nucleus are another source of ipsilateral inputs to mLNTB with their axons showing a significant number of swellings in this area prior to entering LSO.

We also found that a significant number of mLNTB cells respond to contralateral auditory stimulation. The source of this input is unknown but our anatomical observation that these cells can have extensive dendritic trees extending through the MSO may lend a clue. By sending dendrites through the MSO mLNTB cells might have access to the excitatory contralateral spherical bushy cell input that MSO cells receive (Cant, 1991).

We also noted IPSPs in both LNTB subdivisions, and observed synaptic terminals on both cell types that display inhibitory features. Recent data from gerbil SOC brain slices showed that axon collaterals of other LNTB cells may be the source of some of these terminals (Roberts et al., 2014). We have observed that some LSO principal cells send axon collaterals to LNTB cells. LSO cells projecting to the ipsilateral IC can be either glycinergic (inhibitory) or glutamatergic (excitatory) so it is possible that the axon collaterals we observed could be inhibitory (Saint Marie et al., 1989; Glendenning et al., 1992; Oliver et al., 1995).

This unexpected LSO input to pvLNTB is intriguing for other reasons. The LSO is primarily known as the nucleus that generates sensitivity to ILDs, a sound localization cue that becomes important at high frequencies (Tollin, 2003). In contrast, the LNTB has so far mainly been regarded as part of the ITD circuit, considering its projection to the MSO (Cant and Hyson, 1992; Myoga et al., 2014). We did not test pvLNTB neurons for sensitivity to ILD, but our results suggest that these circuits are even more intertwined than previously thought.

### Responses to Sound

Prior to the experiments described here the only information on auditory responses of LNTB cells was from extracellular recordings from cat (Guinan et al., 1972a,b; Tsuchitani, 1977), which used lesions along an electrode penetration to infer the approximate site of recordings made in various SOC regions. Responses to short tones localized to the LNTB region were varied, with most of them being either primary-like, primary-like-with-notch, chopper, onset or phase-locked PSTHs. We have shown that at least some of these can be assigned to a particular cell population. pvLNTB cells are typically primary-like-with-notch but can be primary-like in their response patterns while those in mLNTB can be choppers or onset at CF. Both groups can also show phase-locked responses at low frequencies. Two of the mLNTB cells whose axons headed toward the IC were onset and showed phase-locked spikes to low frequency tones, in one case even generating entrained “high-sync” responses. In mLNTB but not pvLNTB, inhibitory responses to sound were observed.

Many pvLNTB cells are glycinergic (Spirou and Berrebi, 1997) and are thought to project to MSO where, according to one scheme, they would provide an extremely well-timed inhibitory input (Myoga et al., 2014). Our data lend support to this scheme by showing for the first time that these neurons can strongly phase-lock to low-frequency sounds. However, *in vivo* whole-cell recordings from MSO neurons in the same species, combined with pharmacological manipulation provided little support for strong phase-locked ipsilateral inhibition and its hypothesized role in ITD-tuning (Franken et al., 2015).

One of the most striking outcomes of this study is that some mLNTB neurons are sensitive to ITDs of fine-structure or envelope. Only a few extracellular studies in the SOC have reported the recording sites at which ITD-sensitivity was found (Yin and Chan, 1990; Spitzer and Semple, 1995; Pecka et al., 2008; Day and Semple, 2011; Stange et al., 2013). In these studies, the site of recording was often outside the nuclear boundary of the MSO. This was thought to reflect the inherently coarse localization information obtainable from extracellular recording and marking methods, or a propensity toward recording from axons given that somatic spikes in MSO are small. For example, Spitzer and Semple (1995) located many ITD-sensitive neurons near the dorsal pole of the MSO. The data presented here suggest that some of these recordings may actually have been derived from mLNTB neurons.

In contrast to ITD-sensitivity of labeled MSO neurons (Franken et al., 2015), ITD functions of mLNTB neurons displayed broad peaks and could show significant driven spike rates to out-of-phase stimuli (**Figure 14F**). Also, in two cases ITD tuning was stronger or present at lower sound levels for ITDs in the envelope than in the fine-structure of the sound waveform. As previously reported for MSO, ITD-sensitive mLNTB neurons received excitatory phase-locked input to monaural stimuli (judged from EPSPs) and, at least in one case (**Figure 14B**), the phase of the inputs was consistent with the binaural tuning to ITD (**Figure 14A**). This is interesting because monaural phase-locking has been used as one of the criteria for “primary” versus “non-primary” coincidence detection (Goldberg and Brown, 1969; Spitzer and Semple, 1995; Batra et al., 1997). For example, in their classic study in dog, Goldberg and Brown (1969) reported ITD-sensitivity not only in MSO neurons but also MPO neurons. However, in the latter neurons, there was no phase-locking to monaurally evoked responses, which led the authors to conclude that these neurons are not primary coincidence detectors but inherit their ITD-sensitivity from inputs such as the MSO. Whether LNTB neurons create ITD-sensitivity *de novo* or whether they inherit it from MSO neurons remains to be determined: the mere presence of monaural phase-locking, even if predictive of binaural ITD-tuning, does not seem sufficient to us to be indicative of a primary site of coincidence detection.

### Responses to Current

Many cells in the brainstem low-frequency sound-localization pathway including globular and spherical bushy cells, MNTB principal cells and MSO principal cells respond to depolarizing current pulses with a single or a few spikes at current onset. This is due in large part to a low-voltage-activated K channel that, together with other ion channels, allows these cells to respond with a spike output that very precisely replicates their input, a feature needed in a circuit involved with precise timing (Forsythe and Barnes-Davies, 1993; Brown and Kaczmarek, 2011; Golding and Oertel, 2012). In contrast, we observed that LNTB cells responded repetitively for the duration of a current pulse, confirming results *in vitro* (Roberts et al., 2014). It is noteworthy that (m)LNTB cells still had good monaural and binaural temporal properties (showing phase-locking and ITD-sensitivity). In addition the spikes generated in many of these LNTB cells showed a double undershoot as observed *in vitro*, unlike the cell types mentioned above. The combination of repetitive firing to current pulses and strong phase-locking (high VS values) can also be observed in chopper/stellate cells in the cochlear nucleus (Wu and Oertel, 1984; Joris et al., 1994b).

### LNTB Outputs

Recently major emphasis has been placed on the output of LNTB to MSO (Myoga et al., 2014) although the role of inhibition in ITD-tuning is unclear (Roberts et al., 2013; Franken et al., 2015). Not all LNTB neurons project to MSO: in two mLNTB neurons there was sufficient labeling of the axon to determine that they project to IC and not MSO, and others have shown in gerbil and other species that the majority of cells in what is probably mLNTB project primarily to the cochlear nucleus and some to the IC as well (Brunso-Bechtold et al., 1981; Adams, 1983; Nordeen et al., 1983; Covey et al., 1984; Spangler et al., 1987). The study of Cant and Hyson (1992) may have overestimated the number of cells projecting to MSO. In that report, small injections of biocytin into MSO labeled inputs to MSO by retrograde axonal transport. The report verified previous findings that spherical bushy cells from both cochlear nuclei as well as MNTB neurons innervate MSO and also added LNTB as an important source. Many cells were shown to be labeled at all dorsoventral levels of LNTB. Our data suggests that some of the mLNTB cells were labeled in that study not because they project to MSO but because their dendrites run through MSO. Kuwabara and Zook report labeled LNTB cells projecting to MSO, but the two cells shown – recorded in the bat – also innervate LSO (Kuwabara and Zook, 1992). In rodent (Gómez-Álvarez and Saldaña, 2015), LNTB cells do not project to LSO. A recent gerbil *in vitro* study reported labeled LNTB cells with generally small dendritic fields projecting to the MSO, suggesting that they might have been pvLNTB cells (Roberts et al., 2014).

## Conclusion

We show in this paper that the gerbil LNTB includes a population of mLNTB cells that can be separated from pvLNTB cells on anatomical and physiological grounds. Our results emphasize that the role of the LNTB in sound processing is more elaborate than simply providing the ipsilateral inhibitory input to MSO cells and this should be the subject of further study, also considering the prominence of the LNTB in the human SOC (Kulesza, 2008), and its malformation in autism (Kulesza et al., 2011).

## Author Contributions

TF, PS, and PJ designed the study; TF and PS performed research and analyzed data; TF, PS and PJ wrote the manuscript.

## Conflict of Interest Statement

The authors declare that the research was conducted in the absence of any commercial or financial relationships that could be construed as a potential conflict of interest.

## Abbreviations

CF: characteristic frequency
CV: coefficient of variation
EM: electron microscopy
EPSP: excitatory postsynaptic potential
GBC: globular bushy cell
hLNTB: hilar LNTB
ILD: interaural level difference
i.m.: intramuscularly
i.p.: intraperitoneally
IPSP: inhibitory postsynaptic potential
ISI: interspike interval
ITD: interaural time difference
LNTB: lateral nucleus of the trapezoid body
LSO: lateral superior olive
MAC: mitochondrial adherens complex
MNTB: medial nucleus of the trapezoid body
MPO: medial periolivary
MSO: medial superior olive
mLNTB: main LNTB
PFA: paraformaldehyde
PSTH: peristimulus time histogram
pvLNTB: posteroventral LNTB
SOC: superior olivary complex
SPL: sound pressure level
SPON: superior paraolivary nucleus
TB: trapezoid body
VS: vector strength
